# All-Silicon Ultra-Broadband Infrared Light Absorbers

**DOI:** 10.1038/srep38589

**Published:** 2016-12-07

**Authors:** Kazim Gorgulu, Abdullah Gok, Mehmet Yilmaz, Kagan Topalli, Necmi Bıyıklı, Ali K. Okyay

**Affiliations:** 1 Department of Electrical and Electronics Engineering, Bilkent University, Ankara, 06800, Turkey; 2 UNAM-National Nanotechnology Research Center, Bilkent University, Ankara, 06800, Turkey; 3 Institute of Materials Science and Nanotechnology, Bilkent University, Ankara, 06800, Turkey

## Abstract

Absorbing infrared radiation efficiently is important for critical applications such as thermal imaging and infrared spectroscopy. Common infrared absorbing materials are not standard in Si VLSI technology. We demonstrate ultra-broadband mid-infrared absorbers based purely on silicon. Broadband absorption is achieved by the combined effects of free carrier absorption, and vibrational and plasmonic absorption resonances. The absorbers, consisting of periodically arranged silicon gratings, can be fabricated using standard optical lithography and deep reactive ion etching techniques, allowing for cost-effective and wafer-scale fabrication of micro-structures. Absorption wavebands in excess of 15 micrometers (5–20 *μm*) are demonstrated with more than 90% average absorptivity. The structures also exhibit broadband absorption performance even at large angles of incidence (θ = 50°), and independent of polarization.

By exploiting plasmonic structures, electromagnetic energy can be localized into a very small volume and efficient conversion between photons and plasmons can be controlled at sub-wavelength scale^1^,^2^. Noble metals such as gold and silver are commonly used plasmonic materials at visible (VIS) and near-infrared (NIR) ranges. In order to exploit plasmonic enhancement in the mid-wave and long-wave infrared regions, materials with plasma frequencies in this range are required. Common metals have plasma frequencies in VIS and NIR regions. On the other hand, plasma frequency of semiconductors can be modulated by either changing charge carrier concentration (doping) or applying potential gating^3^,^4^. Therefore, highly doped semiconductors emerge as favorable alternatives of metals in plasmonics, especially in the infrared region. The performance of various semiconductor materials has been evaluated in a number of theoretical and experimental studies^5^,^6^,^7^,^8^. Among these semiconductors, silicon has attracted great interest due to silicon integrated photonic devices including sub-wavelength interconnects, modulators, and emission sources^4^,^9^. Silicon also enables chip scale integration at mid-infrared (MID-IR) band where many potential applications arise including chemical and biological sensors, imagers, light sources and spectroscopy^10^.

Demonstration of plasmonic effects by silicon in the infrared region is carried out by others^11^,^12^. Several groups have studied silicon based surface plasmon resonances for bio-chemical sensing applications^13^,^14^,^15^. Frequency selective infrared absorbers have been proposed based on low-resistivity silicon gratings^16^,^17^. For spectroscopy and imaging applications, it is highly desirable to have wideband absorbers. Most of the resonant absorbers suffer from narrow operating absorption waveband. Much of the work in this direction focuses on multi-resonance or tapered metamaterial structures to increase bandwidth^18^,^19^,^20^. One way of increasing absorption band is to use multilayered structures composed of lossy materials absorbing the light gradually. Recently, two studies were reported as in the form of a geometric transition absorber based on metal-dielectric slabs^19^,^20^. Another approach is to combine different resonators to obtain wideband or multiband response 18,21,22. However, as the number of resonators increases, so does the size of a unit cell, the fill factor and the absorption efficiency decrease. Therefore, highly efficient and ultra-wideband absorbers have not previously been proposed or implemented in the MID-IR region. In this study, we theoretically and experimentally demonstrate an ultra-wideband MID-IR absorber. Furthermore, we show that the absorption is related to the free carrier absorption, and vibrational and plasmonic resonances supported by the structure simultaneously. The absorber demonstrated in this study has an enhanced bandwidth without compromising absorption performance by alleviating the issues encountered in the previous studies presented in the literature. More importantly, proposed absorber is based on silicon and it extends silicon photonics to the mid-infrared wavelengths for optoelectronic systems. Such an absorber paves the way to the realization of all-silicon based sensors, imagers and spectroscopy applications, and provides chip-scale integration compatible with CMOS technology.

## Results and Discussion

**Figure 1:**
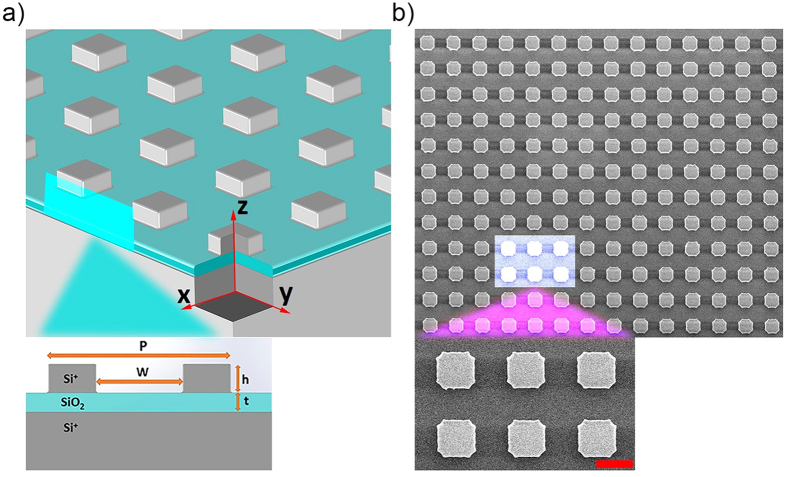
(**a**) Schematic representation of a three layer silicon-silicon dioxide-silicon structure with the top silicon layer patterned as 2D gratings of periodicity P, (**b**) SEM image of one of the fabricated samples with periodicity, P = 8 μm. Scale bar is 4 μm.

Figure 1a presents the schematic diagram of the wideband absorber. The absorber is composed of a three-layer-stack of silicon-silicon dioxide-silicon structure. Only the top silicon layer is patterned periodically along both *x* and *y* directions. For the fabrication of the structures, a commercially available silicon-on-insulator (SOI) wafer is utilized. Conventional optical lithography and deep reactive ion etching (DRIE) techniques are used for the microfabrication of the structures. The thicknesses of the silicon grating and the silicon dioxide layer are h = 1.2 μm and t = 0.8 μm, respectively. Two structures with different periodicities, P = 7 μm and P = 8 μm, and different widths, W = 3 μm and W = 4.2 μm, are implemented. Figure 1b shows the scanning electron microscope (SEM) image of the sample with periodicity, P = 8 μm.

**Figure 2:**
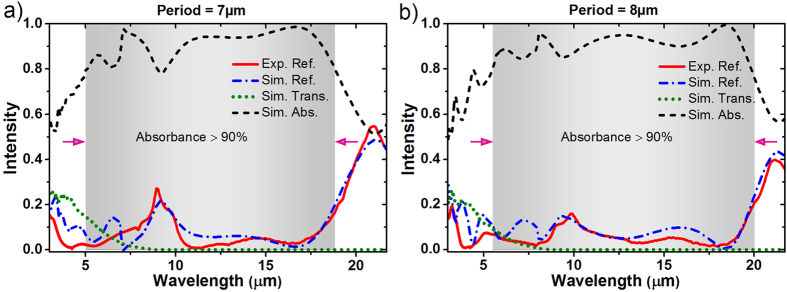
(**a**,**b**) Simulated reflection (Sim. Ref.), transmission (Sim. Trans.) and absorption (Sim. Abs.) spectra and measured reflection (Exp. Ref.) spectrum for the samples with periodicities of P = 7 μm and P = 8 μm.

In order to conduct numerical analysis of these structures, we used finite difference time domain (FDTD) method. Optical properties of highly-doped semiconductors are dominated by electron plasma of the material, and can be modeled using Drude formalism^11^,^12^. The optical properties of the top and bottom silicon layers are extracted using Drude formalism. Our model is confirmed by means of an experimental setup (see Supplementary Information). Doping concentrations of the top (n-type) and bottom (p-type) silicon layers are found to be N_n_ = 5.2 × 10^19^ cm^−3^ and N_p_ = 5.5 × 10^19^ cm^−3^, respectively; and the relaxation frequencies are ω_n_ = 0.04 eV and ω_p_ = 0.07 eV respectively. A characteristic frequency of semiconductors is called plasma frequency, which is defined as the frequency at which the real part of the permittivity vanishes. Plasma wavelengths of the top and bottom silicon layers are extracted to be λ_n_ = 8.28 μm and λ_p_ = 9.42 μm, respectively. In FDTD simulations optical constants of silicon layers are taken from Drude formalism results and the optical constants of silicon dioxide are received from the literature^23^. The absorption in the simulations is defined as A(λ) = 1–R(λ)–T(λ), where T(λ) is the transmitted power and R(λ) is the reflected power. Figure 2 shows the simulated reflection, transmission, and absorption spectra at normal incidence for arbitrarily polarized light. In these computational analyses, transmitted power is calculated at 2.5 μm below the interface between silicon dioxide and bottom silicon layer. Highly-doped silicon has a very high attenuation coefficient (1.61 μm^−1^ at plasma wavelength, λ = 9.42 μm), therefore almost no power is transmitted through the structure beyond plasma wavelength and only a weak transmission is observed at shorter wavelengths from simulation results. Plasma wavelength of the bottom silicon layer can be shifted to shorter wavelengths by increasing the doping density which will further decrease the transmitted power. Two different samples with periodicities of 7 μm and 8 μm provide absorption waveband of 5–18.8 μm and 5.4–20 μm, respectively; yielding excellent absorption performance that is more than 90% in average. Red lines in Fig. 2a-b show the corresponding experimental reflection results of these wideband absorber structures and the measured reflection spectra agree well with the simulated ones.

**Figure 3:**
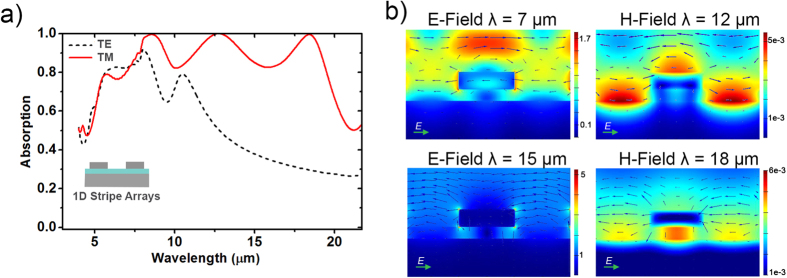
(**a**) Simulated absorption spectra of the 1D structure with periodicity 8 μm and fill ratio of 0.5 for TE and TM polarizations. Inset shows the schematic of 1D silicon – silicon dioxide – silicon structure (**b**) Calculated spatial field distributions for the same structure with periodicity of 8 μm at λ = 7 μm, λ = 12 μm, λ = 15 μm, and λ = 18 μm. The arrows represent the electric field vector.

The effect of multiple resonances can be identified from the absorption peaks in the spectra. In order to understand the nature of absorption processes we first investigated the absorption properties of one dimensional (1D) 1.2 μm-thick silicon stripes on 0.8 μm-thick silicon dioxide spacer on silicon substrate. The periodicity and the fill ratio of the silicon stripe array are 8 μm and 0.5, respectively. Figure 3a shows the simulated normal incidence absorption spectra of this 1D structure for both TE (E-field is parallel to the silicon grating direction) and TM (E-field is perpendicular to the silicon grating direction) polarization configurations. For TE polarization, absorption between 6 μm and 9 μm is dominated by free carrier absorption in heavily doped silicon layers. This is the classical Drude absorption by free carriers and can be spectrally tuned by controlling the carrier concentration of silicon layers. Since the real part of the permittivity is very low around the plasma frequency, light can easily penetrate into the material. Therefore, reflection minimum and absorption maximum of a non-corrugated highly doped silicon occurs near the plasma frequency (see Supplementary Fig. S3). Around 10 μm, silicon dioxide exhibits molecular resonance due to Si-O-Si vibrations. Asymmetric stretching vibration of Si-O-Si bridges contributes to the absorption in this region^24^. At longer wavelengths, free carrier conduction dominates, thus the reflectivity increases and the absorption decreases. For TM polarization, absorption spectrum gives the evidence for the excitation of surface plasmon modes. Two additional absorption peaks are observed at longer wavelengths (at 12.7 μm and 18 μm) in addition to the absorption bands at shorter wavelengths.

Additional information can be obtained from spatial field distributions. Figure 3b shows the calculated field distributions at four major points in the absorption spectrum of the structure with periodicity, P = 8 μm using the FDTD method. The electric field at 7 μm is mainly concentrated on the top of the silicon grating, where incident field constructively interferes with the reflected field. At this range, field also penetrates through the silicon layers and is absorbed by free carriers. At wavelengths longer than the plasma wavelength, highly doped silicon layers behave like metals and the structure exhibits the properties of the common metal/insulator/metal (MIM) structures. Similar MIM structures consisting of a metal structure and a metal film separated by a dielectric layer were reported by several groups^25^,^26^,^27^,^28^,^29^,^30^,^31^. These studies have shown the coupling between different plasmonic modes and investigated the effects of the structure periodicity, the fill ratio and the dielectric thickness on these plasmonic modes. When the periodicity of the structure is on the order of the wavelength, propagating surface plasmon resonances can be generated^25^,^28^,^30^. The magnetic field distribution at 12 μm shows strong confinement at the top of the silicon gratings and between two adjacent gratings. The field distribution at this range confirms that the absorption at this point arises from grating induced silica-side propagating surface plasmons (PSP). On the other hand, magnetic field at 18 μm is concentrated below the silicon gratings, in the gap filled by silicon dioxide. The absorption peak at this point is due to the gap-plasmon (GP) modes supported by the cavity between silicon layers as reported for similar MIM structures^25^,^26^,^27^,^28^,^29^,^30^,^31^. Electric field distribution at 15 μm shows strong confinement at the top and bottom of the silicon grating. Both PSP and GP modes have broad absorption bands (more than 5 μm), hence both of these modes have partial effects on the absorption at 15 μm.

**Figure 4:**
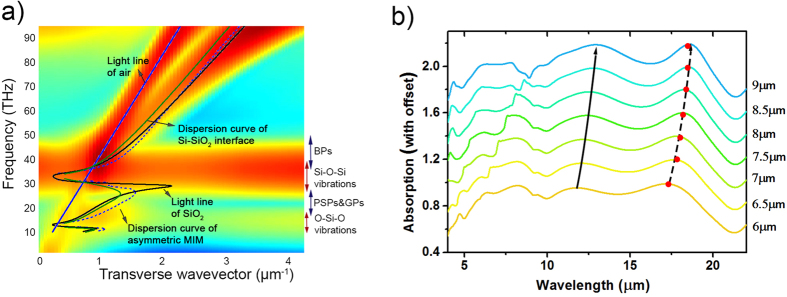
(**a**) Simulated and analytical dispersion relation for the silicon-silicon dioxide-silicon-air structure. Abbr: BPs (Bulk Plasmons), PSPs (Propagating Surface Plasmons), GPs (Gap Plasmons). (**b**) Dependence of the absorption spectrum on the structure periodicity. Fill ratio is kept constant as 0.5 and the periodicity is varied from 6 μm to 9 μm.

Dispersion relation of the surface waves supported by unpatterned Si-SiO_2_-Si-Air structure is calculated by using a bandstructure technique of the FDTD method. In this bandstructure technique we do a parameter sweep over the transverse wavevector and look for frequencies with strong resonances. Simulated dispersion relation of this multilayer system is shown in Fig. 4a. Two vibrational bands of silicon dioxide, located at 13.6 THz (22 μm) and 30 THz (10 μm)^24^, can be observed. Analytical dispersion relation of Metal/Insulator/Metal like structures are reported previously^32^,^33^. The transverse magnetic plasmon dispersion relation for an asymmetric MIM structure is given by,


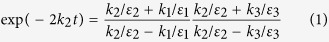


where *ε*_*1*_, *ε*_*2*_, and *ε*_*3*_represent the permittivities of the layers; bottom Si^+^, SiO_2_, and top Si^+^, respectively. *t* is the gap thickness. *k*_*1*_, *k*_*2*_, and *k*_*3*_ are the wavevectors perpendicular to the interfaces defined by momentum conservation:


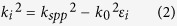


for *i* = 1, 2, and 3. *k*_*spp*_ is the wavevector of the surface plasmons. Surface plasmon polariton dispersion relation at a single interface is given by^34^:


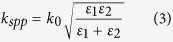


Dispersion relation of MIM systems are implicit functions and can be solved numerically. Dashed blue line in Fig. 4a show the theoretical result for the dispersion relation of silicon-silicon dioxide-silicon structure. Solid black line in the same figure represents the dispersion relation of propagating surface plasmons between silicon and silica. The plasmonic resonances, PSPs and GPs, are excited in the frequency range between the two vibrational bands of the silicon dioxide.

Figure 4b shows the simulated absorption spectra for various grating periodicities. In these simulations the fill ratio is kept constant as 0.5 and the periodicity is varied from 6 μm to 9 μm. Both of the plasmonic resonances show redshift with increasing periodicity. PSP mode (solid black line) is excited by periodic nature of the structure, so the resonance wavelength redshifts with increasing periodicity as expected. However, the resonance shift of GP mode essentially depends on the width of the silicon gratings rather than the periodicity. The constructive interferences of GP mode, reflected back and forth at the edges of the cube, create a cavity resonance as in a Fabry–Perot interferometer. Therefore, the width of the silicon grating plays an essential role for the resonance shift as being a parameter of this cavity^25^,^28^,^30^,^31^. GP resonance wavelength is related to the width of the silicon grating, *w,* by^25^,^30^,^32^.


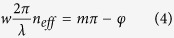


where *n*_*eff*_ is the effective index of the gap and can be calculated from dispersion relation, *m* is the mode order and *φ* is the phase of the reflection coefficient of the mode at the end of the gap. The reflection phase *φ,* zero for a perfect Fabry-Perot cavity, is nonzero for a finite waveguide and depends on the structure and material properties^26^,^31^. There is no analytical solution for reflection phase, hence it can be computed numerically or it is estimated as an average value minimizing the error of resonance wavelengths^25^,^26^,^30^,^31^. In our structure we observe that the change in the reflection phase is nonsignificant when the fill ratio is kept constant. In order to determine reflection phase, we used one of the simulation results and we extracted the reflection phase as 63° according to the resonance wavelength in the simulation result. Since the reflection phase does not change significantly, all the other resonance wavelengths can be estimated accurately by Eq. 4 for the first order resonance (m = 1). As shown in Fig. 4b the simulated resonance wavelengths (dashed black line) almost match up with semi-analytically calculated (red circles) ones with an error less than 0.5%.

**Figure 5:**
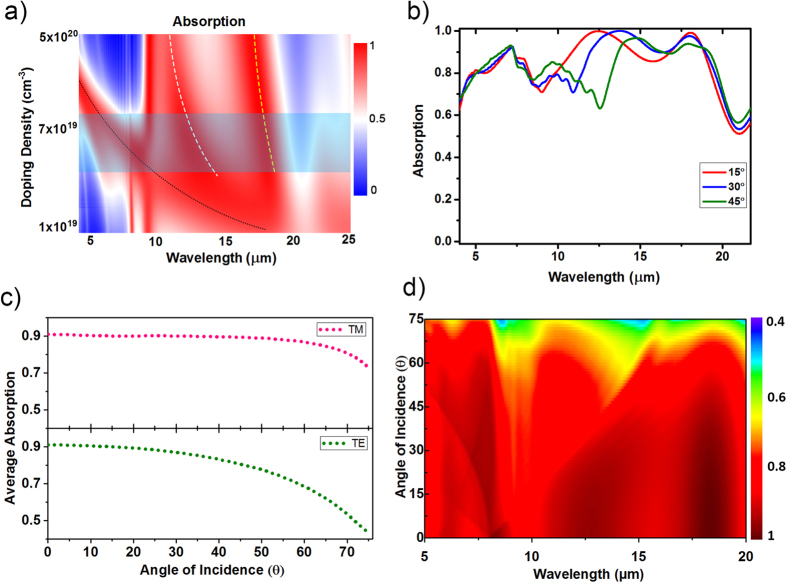
(**a**) Absorption spectrum as a function of wavelength and doping density. (**b**) Absorption spectrum of the structure with periodicity 8 μm and fill ratio 0.5 for three different incidence angles. The structure is illuminated by a TM polarized plane wave. (**c**) Average absorption as a function of angle of incidence for TM and TE polarizations. (**d**) Absorption spectrum as a function of wavelength and angle of incidence.

We also investigated the performance of the structure with periodicity 8 μm and fill ratio 0.5 for different doping concentrations. Figure 5a shows absorption spectrum as a function of doping density and wavelength. Dotted black line in the figure shows how plasma frequency shifts with increasing doping density. Due to the high penetration of field near the plasma frequency there exists an absorption band following the plasma frequency line. Plasmonic absorption bands (dashed white and dashed yellow lines) also shift to shorter wavelengths with increasing doping density. Blueshift of plasmonic absorption bands can be confirmed by dispersion relations. Increasing doping concentration shifts dispersion curves to higher frequencies, thus excitation of plasmonic resonances occurs at shorter wavelengths (See Supplementary Fig. S4). The blue rectangle shows the doping concentration range where ultra-broad absorption band can be obtained for this structure. Doping concentrations of silicon layers in the experimental part fall into this region.

In order to enrich our study and investigate further the origin of the plasmonic resonances we conducted angle-dependent simulations using the FDTD method. Figure 5b shows the evolution of the absorption spectra with increasing incidence angle. As shown in the figure, one of the plasmonic modes redshifts with the increasing incidence angle. The dependence of the plasmonic mode on the angle of incidence indicates that this is a propagating mode. As this resonance point shifts to longer wavelengths, vibrational absorption band of silicon dioxide becomes discernable around 10 μm. On the other hand, the other plasmonic mode around 18 μm as being a GP mode is almost independent of the incidence angle of the light. This is in agreement with previous studies done on similar systems by other groups^25^,^28^,^31^. Nearly all broadband absorbers are designed to absorb light independent of polarization and angle of incidence. In order to achieve a polarization-independent electromagnetic response we design our structure to be symmetric in both *x* and *y* directions. Figure 5c shows the average absorption as a function of incidence angle for both TM (x-polarized, θ is in x-z plane) and TE (y-polarized, θ is in x-z plane) polarizations. At normal incidence, the absorption spectra for both polarizations are the same because of the symmetry in the structure. For TM polarization average absorption is above 80% even at 70° angle of incidence and then it decreases dramatically. For TE polarization absorption is maintained around 80% at up to 50° angle of incidence. Figure 5d shows the extinction spectra as a function of wavelength and angle of incidence for the light composed of both TM and TE polarization components. As it is seen in the figure, the broadband response is maintained when the incidence angle smaller than 50°, which is a significant achievement for resonant absorbers.

## Conclusion

In conclusion, we have demonstrated ultra-broadband MID-IR absorbers on low-resistivity silicon. Periodically arranged silicon gratings are fabricated by using standard optical lithography and DRIE of silicon allowing for a cost-effective fabrication of micro-scale structures. Two different structures with periodicities of 7 μm and 8 μm provide average absorptivity of more than 90% in the waveband of 5–18.8 μm and 5.4–20 μm, respectively, with a total thickness less than the quarter of the wavelength. The structures also exhibit broadband absorption performance even at high angles of incidence (θ = 50°) independent of the polarization. Our detailed analysis indicates that ultra-broadband light absorption originates from superposition of the free carrier absorption, and vibrational and plasmonic resonances excited at different portions of the MID-IR range. Silicon-based strong and ultra-broadband absorption of light at MID-IR region is very promising for realization of silicon based infrared imagers, sensors, and enhanced spectroscopy. Combined with extensive processing and device design knowledge base of silicon, presented absorbers yield fabrication simplicity and CMOS compatible optoelectronic integration.

## Methods

### Device fabrication and measurement

Fabrication of our structures starts with commercial SOI wafers consisting of 500 μm thick highly doped handle layer silicon substrate at the bottom with resistivity range of ρ = 0.002–0.005 Ω-cm, 800 nm thick silicon dioxide in the middle and 2 μm thick highly doped device layer silicon at the top with resistivity range of ρ = 0.001–0.0015 Ω-cm. We first thinned the device layer silicon from 2 μm to 1.2 μm by using an SF_6_ based isotropic RIE process. After thinning the device layer of the SOI to the desired thickness of 1.2 μm, we used standard photolithography procedures to define the desired patterns. First, we spun a positive photoresist (AZ 5214E) at 4000 r.p.m. and baked the photoresist at 110 °C for 50 seconds. Then, we exposed the photoresist with a contact aligner and developed the photoresist with DI water diluted AZ 400 K developer. Using a BOSCH process recipe that is SF_6_ based RIE at the etch step, and C_4_F_8_ based deposition at the passivation step, the 1.2 μm thick Si is patterned till the 800 nm silicon dioxide surface is reached. Then, the remaining photoresist is removed by an oxygen plasma cleaning recipe. In order to measure the reflection of the fabricated structures, Bruker HYPERION 2000 IR microscope and Bruker Vertex 70 v Fourier transform infrared (FTIR) spectrometer are used. The measurements are referenced to a gold-coated flat silicon mirror.

### Simulations

Electromagnetic wave simulations are performed using FDTD Solutions by Lumerical Inc, a commercially available FDTD simulation software package. For 3D simulation setup; on the x and y axes periodic boundary conditions are used, and on the z-axis (propagation direction) a perfectly matched layer (PML) is used. For 2D simulation setup; on the x-axis periodic boundary condition is used, and on the y-axis (propagation direction) PML is used. In all of the simulations, we employed cubic mesh with a mesh size of 10 nm.

## Additional Information

**How to cite this article**: Gorgulu, K. *et al*. All-Silicon Ultra-Broadband Infrared Light Absorbers. *Sci. Rep.* **6**, 38589; doi: 10.1038/srep38589 (2016).

**Publisher's note:** Springer Nature remains neutral with regard to jurisdictional claims in published maps and institutional affiliations.

## Supplementary Material

Supplementary Information
