# Supplementary material for: All-Silicon Ultra-Broadband Infrared Light Absorbers
Source: Sci Rep. 2016 Dec 7;6:38589. doi: 10.1038/srep38589 (PMC5141492; doi:10.1038/srep38589)
Supplement: Supplementary Information [file srep38589-s1.pdf]

All-Silicon Ultra-Broadband Infrared Light Absorbers

Supplementary Information

Kazim Gorgulu, Abdullah Gok, Mehmet Yilmaz, Kagan Topalli, Necmi Bıyıklı, and Ali K. Okyay

Optical Characterization

In order to determine optical properties of the highly doped silicon, Drude formalism is used:

$$\varepsilon(\omega) = \varepsilon_\infty \left(1 - \frac{\omega_p^2}{\omega\left(\omega + \frac{j}{\tau}\right)}\right)$$

where ε_∞ is the permittivity value for $\omega >> \omega_p$ and taken as 11.7, τ is the relaxation time, ω_p is the plasma frequency:

$$\omega_p^2 = \frac{Ne^2}{\varepsilon_0 \varepsilon_\infty m^* m_0}$$

where N is the free carrier concentration per cm^3, e is the elementary charge, ε_0 is the vacuum permittivity, m^* is the effective mass, and m_0 is the electron mass. The procured SOI wafer has n-type silicon layer at the top and p-type silicon layer as the substrate. Resistivity values of these silicon layers correspond to the doping density on the order of $N = 5 \times 10^{19}\ cm^{-3}$ [1]. In order to extract optical properties of both the n-type and p-type silicon layers, we consider plasma frequency and relaxation time as our fitting parameters and we start calculations with $N = 5 \times 10^{19}\ cm^{-3}$ and $1/\tau = 0.037$ (eV) [2]. We optimized these parameters by fitting FDTD calculations to our experimental results. Eventually we obtained best fit for $N_n = 5.2 \times 10^{19}\ cm^{-3}$ and, $N_p = 5.5 \times 10^{19}\ cm^{-3}$ for n-type and p-type silicon layers, respectively. These carrier concentration values correspond to plasma wavelengths of $\lambda_n^{pl} = 8.28\ \mu m$ and $\lambda_p^{pl} = 9.42\ \mu m$ for n-type and p-type silicon layers, respectively. Supplementary Figure S1 shows the modeled real and imaginary permittivity values of our n-type and p-type silicon layers. Supplementary Figure S2 shows the measured, simulated and also analytically calculated (T-matrix formalism) reflection results of the SOI structure without patterning. In numerical calculations, optical constants of silicon dioxide are taken from the literature [3]. As seen in this figure the numerical and the analytical reflection spectra fit very well to the experimental reflection spectrum indicating validity of our model.

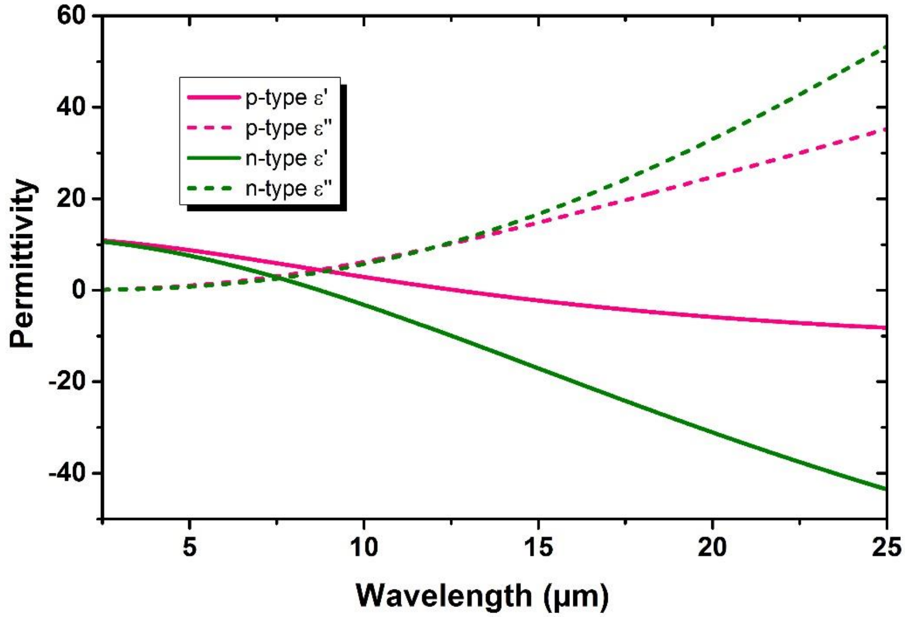

Supplementary Figure S1. Real and imaginary parts of the permittivity for heavily doped n-type and p-type silicon for top and bottom silicon layers of our structure.

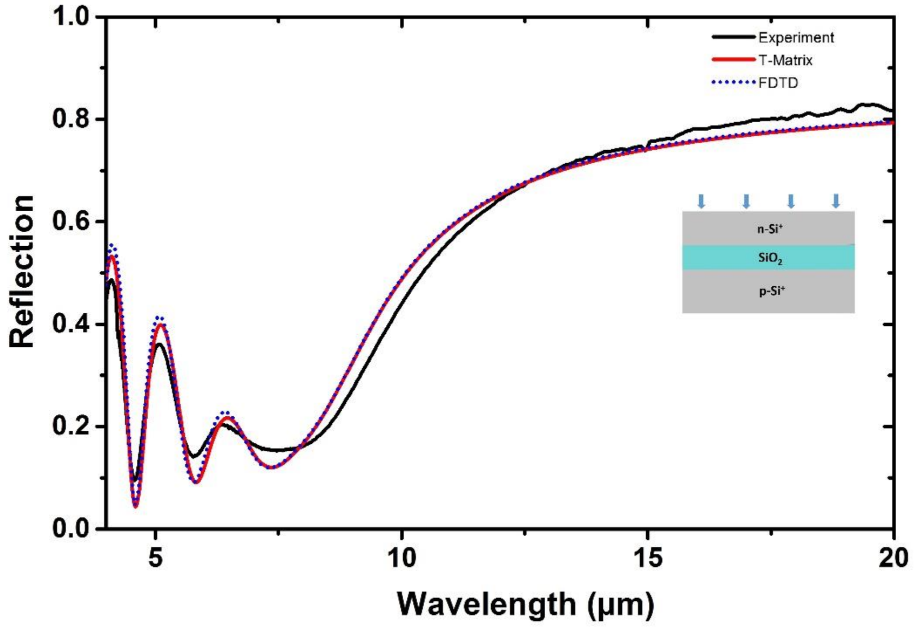

Supplementary Figure S2. Measured, simulated and analytically calculated (T-matrix) reflection spectrum of the SOI structure. Inset illustrates the SOI structure.

Free Carrier Absorption

Highly doped silicon behaves differently at low and high frequencies. In the low frequency regime as $\omega \Rightarrow 0$, $1/\omega$ term dominates and imaginary part of the permittivity increases. Thus, at low frequencies, material behaves like a perfect conductor (e.g. a metal). In the high frequency regime $1/\omega^2$term dominates and imaginary part of the permittivity vanishes. Thus at high frequencies, the optical properties of the highly doped silicon are like those of insulators. Between low and high frequency regimes there is a characteristic transition frequency called plasma frequency at which material's optical response changes from metallic to dielectric. At the plasma frequency, material's real part of the permittivity vanishes, but material's imaginary part of the permittivity is still finite. Around the plasma frequency, since the real part of the permittivity is very low, light can easily penetrate through the material and it is absorbed due to the finite absorption coefficient. Figure S3 shows the bulk absorption of the 4 μm thick silicon with different doping concentrations. As it seen in the graph, absorption peak blueshifts as the concentration increases.

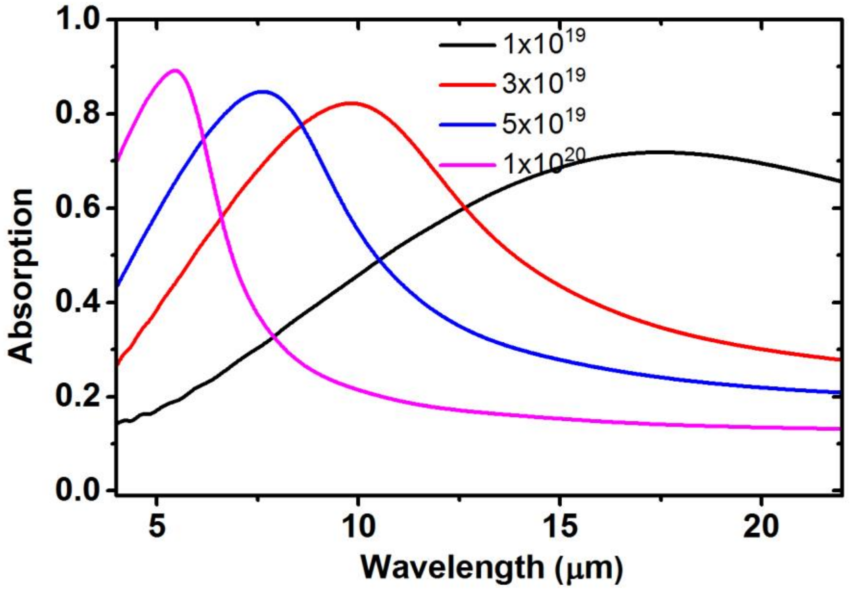

Supplementary Figure S3. Simulated absorption spectra of 4 μm thick silicon with different doping concentrations.

Dispersion relation for different doping concentrations

Simulated dispersion relations of the non-corrugated silicon-silicon dioxide-silicon-air structure for different doping concentrations are shown in Figure S4 (a,b,c). Two vibrational bands of silicon dioxide are at 13.6 THz (22 μm) and 30 THz (10 μm). When the doping concentration is $3x10^{20}$ we can observe an additional absorption band around the plasma frequency. As the doping concentration decreases to $1x10^{20}$ and $5x10^{19}$ the absorption band at plasma frequency region merges with the vibrational band of silicon dioxide. Analytical solution of the dispersion relation for three different concentration are also shown in Figure S4 (d).

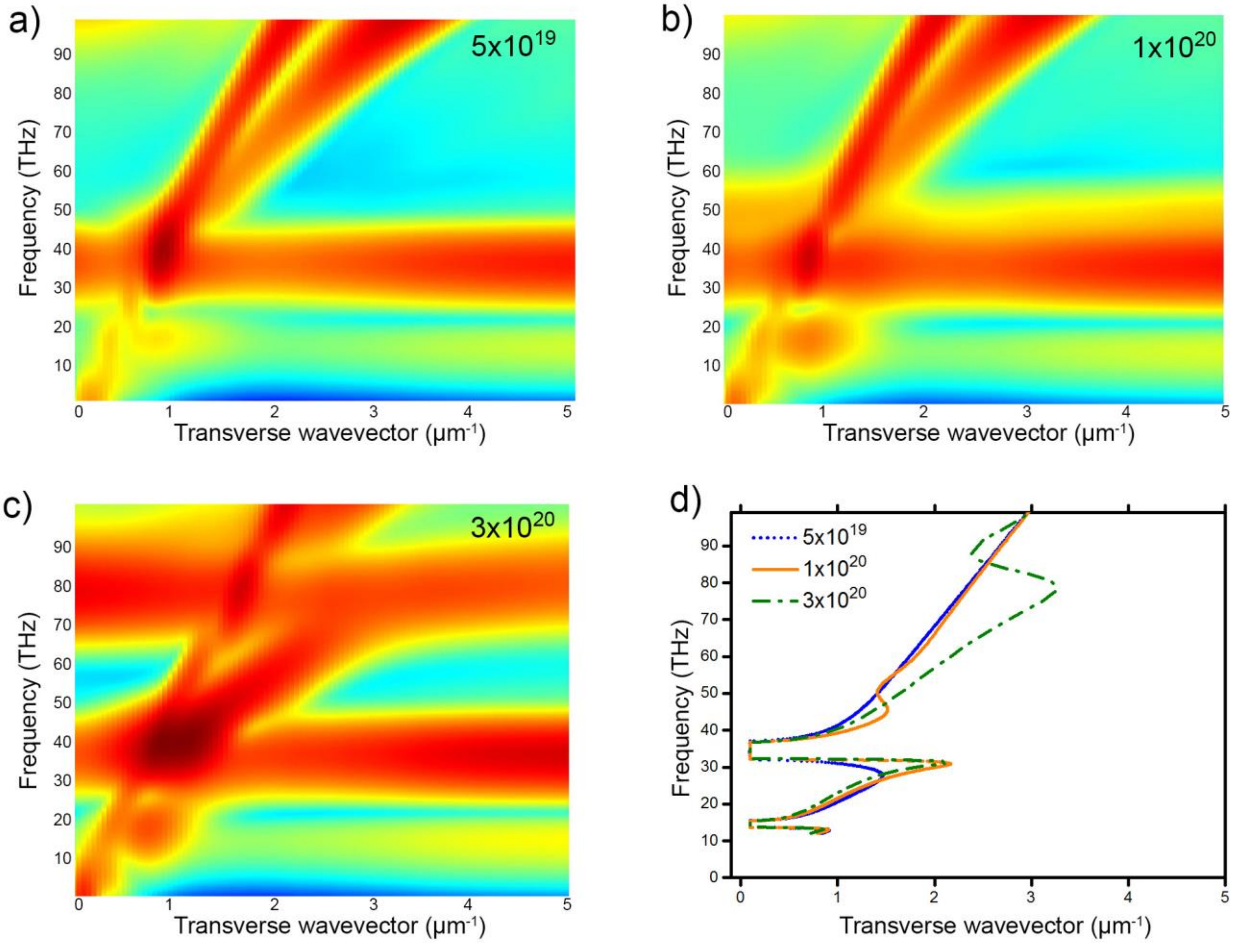

Supplementary Figure S4. (a,b,c) Simulated dispersion relations of the silicon-silicon dioxide-silicon-air structure for the doping concentrations of $5x10^{19}$ cm^{-3}, $1x10^{20}$ cm^{-3} and $3x10^{20}$ $cm^{-3.}$(d) Analytically calculated dispersion relations of the MIM structure for the doping concentrations of $5x10^{19}$ cm^{-3}, $1x10^{20}$ cm^{-3} and $3x10^{20}$ $cm^{-3.}$.

References

Pierret R. F. *Semiconductor Device Fundamentals*; Reading, MA: Addison-Wesley, 1996.

Cleary, J. W.; Peale, R. E.; Shelton, D. J.; Boreman, G. D.; Smith, C. W.; Ishigami, M.; Buchwald, W. R. IR permittivities for silicides and doped silicon. JOSA B 2010, 27, 730-734.

Palik E. D. *Handbook of Optical Constants of Solids*; New York: Academic Press, 1985.
